# A Comparative Study of Routing Protocols of Heterogeneous Wireless Sensor Networks

**DOI:** 10.1155/2014/415415

**Published:** 2014-06-22

**Authors:** Guangjie Han, Xu Jiang, Aihua Qian, Joel J. P. C. Rodrigues, Long Cheng

**Affiliations:** ^1^Department of Information & Communication Systems, Hohai University, Changzhou 213022, China; ^2^Changzhou Key Laboratory of Photovoltaic System Integration and Production Equipment Technology, Changzhou 213022, China; ^3^Institute of Telecommunications, University of Beira Interior, 6201-001 Covilhã, Portugal; ^4^Department of Computer and Communication Engineering, Northeastern University, Qinhuangdao 066004, China

## Abstract

Recently, heterogeneous wireless sensor network (HWSN) routing protocols have drawn more and more attention. Various HWSN routing protocols have been proposed to improve the performance of HWSNs. Among these protocols, hierarchical HWSN routing protocols can improve the performance of the network significantly. In this paper, we will evaluate three hierarchical HWSN protocols proposed recently—EDFCM, MCR, and EEPCA—together with two previous classical routing protocols—LEACH and SEP. We mainly focus on the round of the first node dies (also called the stable period) and the number of packets sent to sink, which is an important aspect to evaluate the monitoring ability of a protocol. We conduct a lot of experiments and simulations on Matlab to analyze the performance of the five routing protocols.

## 1. Introduction

### 1.1. Background and Motivation

Wireless sensor networks (WSNs) have been applied in many fields in recent years, because the sensor nodes can be deployed without any infrastructure and the network can monitor many dangerous or remote places that people cannot reach. At the same time, many of the latest researches related to WSNs mainly focused on routing, coverage, and localization [1]. WSNs are characterized with low-cost microsensor nodes with wireless capability, low power consumption for transmitting data, resource constraints, and battery energy limitation. Since sensor nodes have limited energy, it is urgent to introduce the energy-saving techniques in order to extend the lifetime of WSNs.

To pursue the effective routing protocols for WSNs, many researchers have done lots of studies recently and got the result that a scheme with hierarchy and clustering is promising in improving the scalability and extending the lifetime of WSNs. Low-energy adaptive clustering hierarchy (LEACH) [2] protocol is a classical protocol. Clustering is an efficient method to handle scalability problem and energy consumption challenge. For this reason, it is widely exploited in WSN applications [3].

To further prolong the lifetime of the network and make WSNs more suitable for various scenarios, some researchers proposed WSNs with heterogeneity [4]. Theoretically, we can divide HWSNs into two categories: one is that sensor nodes are deployed with different communication radius [5] and the other is that sensor nodes are deployed with different energy [6]. In fact, heterogeneous routing protocols are very common in WSNs routing protocols. Heterogeneous routing protocols should satisfy the following properties [7].

- *Balancing Energy Consumption.* The energies of the nodes in the network are different from each other when the nodes are deployed in the network for the first time. Because of restricted energy resource and large number of deployed sensor nodes, changing the battery for the nodes is a very tough work and sometimes is impossible in some particular scenarios. Then, we deploy some nodes with more energy in the network to act as the center of data aggregation, processing, and transmission so that the energy dissipation of the whole network can be balanced.
- *The Coordination of Communications.* The communication environment in some sensing areas is unfavorable due to the obstacles, so deploying the nodes with different communication radius is sometimes necessary.
- *Effectiveness for Computation and Storage.* The computational and storage capability of a sensor node is very limited. In some protocols, nodes have to regularly act as the aggregation and relay nodes, and it is necessary for these nodes to have better computational and storage ability than the other nodes to meet this requirement.

### 1.2. Contributions

In comparison with the homogeneous WSNs, the latest proposed HWSN routing protocols have tried to extend the lifetime of network, prolong the stable period, and achieve the reliable data transmission by deploying the sensor nodes with different capabilities. However, the proposed protocols often cannot balance the energy consumption of sensor nodes efficiently. It is vital to minimize and balance the energy consumption among sensor nodes in a network to improve its performance. In this paper, we will compare the performance of three proposed HWSN protocols recently together with two classical protocols in the same HWSN model. These five protocols are all cluster-based. This paper aims to analyze which protocol can outperform others through comparisons under various scenarios, which will lead us to propose more energy-efficient protocols in HWSNs.

### 1.3. Paper Organization

The remainder of this paper is organized as follows. In Section 2, related works are briefly introduced. In Section 3, the protocols we compare are presented in detail. In Section 4, we will show you the specific HWSN model in a Matlab platform. In Section 5, simulations of the protocols and the results are given. In Section 6, we summarize the paper and give the future work.

## 2. Related Work

Recently, many WSN routing protocols have been proposed to improve the performance of the network [8, 9]. They can be divided into two categories: cluster-based protocols [6, 10, 11] and plain-based protocols [5, 12, 13]. As we all know that LEACH is proposed based on the homogeneous WSNs, while, in the practical applications, heterogeneity of nodes cannot be avoided. Proposing the protocol which is suitable for HWSNs is needed. When LEACH is utilized in HWSNs, every sensor node has to select a random number. If the number is less than a threshold *T*(*n*), the sensor node becomes a CH for the current round. The threshold is set as follows:

(1) $$\begin{array}{c} T\left(n\right)=\{\begin{array}{cc} \frac{p}{1-p*\left(r\mathrm{mod}1/p\right)}, & \text{if}\;s\in G\\ 0, & \text{otherwise}, \end{array} \end{array}$$

where *p* is the proper percentage of CH nodes in the whole WSN, *r* is the current round of election, and *G* is the set of nodes that are not elected to be CH. Many LEACH-type schemes are applied in homogeneous WSNs. In homogenous sensor networks, sensor nodes cannot adapt well to the presence of heterogeneity when the network is in operation. As a result, these nodes which consume more energy will die first, and as a result the LEACH-type protocols turn out to be unstable. On the whole, there are lots of specific WSN applications that could highly benefit from being equipped with a percentage of the nodes which have more initial energy than the normal nodes, because these nodes make sure that there is more stable or dependable feedback from the network. And, in some cases, the stable period is a very important concern.

The proposed cluster-based routing protocols to handle heterogeneity in WSNs mainly focus on three aspects: (1) electing the cluster head by the energy prediction scheme; (2) saving energy consumption by multihop between cluster head and sink; (3) using the evolutionary algorithms.

### 2.1. The Energy Prediction Scheme

To get better performance, stable election protocol (SEP) [6] is proposed to maintain the hierarchical routing in the HWSNs where two types of nodes have their own election probability. Distributed energy-efficient clustering (DEEC) assumes that a WSN with two types of nodes of different initial energy levels is a two-level heterogeneous network, and the one with three types of nodes of different initial energy levels is a three-level heterogeneous network [14]. In DEEC, the probability for a node to be a CH is based on the ratio between the residual energy of the node and the average energy of the whole network. So the node with more initial energy and residual energy is more likely to be elected as a CH. Other prediction-based cluster schemes include energy dissipation forecast and clustering management (EDFCM) [15], which is an improvement of DEEC, reliable routing based on energy prediction (REP) [16], and energy-efficient prediction clustering algorithm (EEPCA) [17].

### 2.2. Multihop Transmission

**Figure 1:**
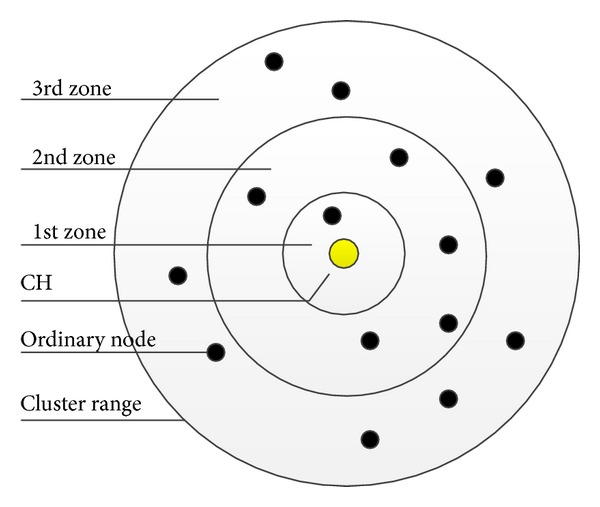
An example of cluster reorganization.

In [18], Younis and Fahmy proposed the hybrid energy-efficient distributed (HEED) clustering algorithm for HWSNs. HEED combines communication range limits and intracluster communication consumption information to improve LEACH protocol. Every sensor node has the initial probability to become a tentative CH depending on its residual energy, and the final CH is selected according to the consumption information. In HEED, the cluster heads are randomly deployed in the sensing area, which makes HEED a cluster-based protocol whose CHs are dynamically selected. HEED has the following advantages: (1) maximizing network lifetime; (2) minimizing control overhead; (3) improving the stability of data transmission; (4) selecting well-distributed cluster heads and well-knit clusters. However, HEED fails to take the balanced energy dissipation among CHs into account. Those CHs near the sink consume energy more quickly than others and they would die first, which causes the energy hole around the sink [19]. This energy hole is a common phenomenon in this kind of networks where there are many CHs transmitting the information to only one sink node. To maximize the lifetime of the network, Senouci et al. proposed the EHEED (extended HEED) [20]. The procedure of selecting CHs is the same as that of HEED, but the way to save energy of EHEED is based on two lemmas which are proved by [21] so as to build a multihop path to the sink. The main idea of these two lemmas is trying to find a proper relay node A from the ordinary nodes within the cluster which resides between node B and node C during the data transmission phase. To find the proper node A, the communication range of the cluster is evenly divided into three zones to compute the parameters. The example of this kind of cluster is shown in Figure 1.

In [22], Kumar et al. proposed a stable election clustering protocol called energy-efficient heterogeneous clustered (EEHC) scheme in the heterogeneous model. The nodes in the network are divided into three categories according to their initial energy: the normal nodes, the advanced nodes, and the super nodes. Apparently, the normal nodes have the least energy, the advanced nodes have more energy than the normal ones, and the super nodes have the highest level of energy. EEHC is based on SEP, and the three types of nodes in EEHC have their own election probability to be CHs within a fixed time to keep stable. In [11], Kumar et al. improved EEHC further and proposed a multihop clustering protocol called MCR. In MCR, the multihop path is built to reduce the energy consumption.

### 2.3. The Improvement of Evolutionary Algorithms

Researchers combined the cluster scheme with the biologically inspired routing scheme, and they proposed the evolutionary algorithms (EAs). The EAs are used to handle the cluster-based problem to optimize energy consumption and prolong lifetime of network with heterogeneity, such as energy-aware evolutionary routing protocol (EAERP) [23], evolutionary-based clustered routing protocol (ERP) [24], and stable-aware evolutionary routing protocol (SAERP) [25]. The evolutionary-based routing protocol EAERP redesigned some significant features of EAs, which can assure longer stable period and extend the lifetime with efficient energy dissipation. The protocol ERP overcame the shortcomings of hierarchical clustering-algorithm-based genetic algorithm [26] by uniting the clustering aspects of cohesion and separation error, and then a new fitness function was proposed based on these two clustering aspects. The fitness function is the primary factor used to minimize network energy consumption. SAERP combined the main idea of SEP and EAs, and SAERP mainly aimed at increasing the stability of the network. So these routing schemes which are inspired genetically demonstrated their advantages in prolonging the lifetime of HWSNs.

Table 1 summarizes all the routing protocols above, and some performances of them are compared simply.

## 3. Typical Protocols for HWSNs

In this section, three latest typical cluster-based HWSN protocols are introduced in detail. They are energy dissipation forecast and clustering management (EDFCM), multihop communication routing (MCR) protocol, and energy-efficient prediction clustering algorithm (EEPCA). These three protocols are all hierarchical, and they represent three kinds of cluster-based techniques which are used for heterogeneous wireless sensor networks to prolong the lifetime of the network.

### 3.1. EDFCM

In [15], Zhou et al. proposed a new model for HWSNs with new energy and computation heterogeneity. By using a mathematical method, the authors acquire the energy dissipation model and the priority percentage of cluster heads in HWSNs. In addition, to improve the cluster-based scheme in LEACH-type protocols, a new type of energy-efficient protocol EDFCM which can guarantee the reliable transmission in HWSNs is proposed. CHs selection of this protocol is based on an energy dissipation forecast method and a clustering management method. EDFCM takes the remaining energy and consumption rate of all nodes into account.

#### 3.1.1. The Algorithm of Cluster Head Selection

To predict the energy consumption in the next round, the average energy consumption of CHs of the two types of nodes in previous round is used. If a node has higher forecasted residual energy which is based on the previous prediction value of energy consumption, it will be selected as a CH. In EDFCM, these two types of nodes are set to be type 0 and type 1 with different levels of energy. The weighted probabilities for the two types of nodes to be selected as CHs are defined as

(2) $$\begin{array}{c} P_{i}\left(r+1\right)=\{\begin{array}{c} \frac{p}{1+\alpha m}\times \frac{E_{i}\left(r\right)-E_{Pr\_T0}\left(r\right)}{\bar{E}\left(r+1\right)},\\ \text{if}\;\text{type}\;0\\ \frac{p}{1+\alpha m}\times \left(1+\alpha \right)\frac{E_{i}\left(r\right)-E_{Pr\_T1}\left(r\right)}{\bar{E}\left(r+1\right)},\\ \text{if}\;\text{type}\;1, \end{array} \end{array}$$

where *E*_*i*_(*r*) is the residual energy of node *i* in round *r*, *E*_*Pr*_*T*0_(*r*) and *E*_*Pr*_*T*1_(*r*) are the average energy dissipations of these two types of cluster heads in the *r* round, respectively, $\bar{E}(r+1)$ is the average energy of nodes in *r* + 1 round, and

(3) $$\begin{array}{c} \bar{E}\left(r+1\right)=\frac{1}{N}\times E_{\text{total}}\times \left(1-\frac{r+1}{R}\right), \end{array}$$

where *E*_total_ is the total initial energy of all nodes in the network and *R* is an estimated round of lifetime of the whole network, which is defined as

(4) $$\begin{array}{c} E_{\text{total}}=N\times E_{0}\times \left(1+\alpha m\right),\\ R=\frac{E_{\text{total}}}{E_{\text{round}\_\text{total}}}, \end{array}$$

where *E*_round_total_ is the total energy consumption in a round.

#### 3.1.2. Operation Mechanism of EDFCM

The operation of EDFCM protocol includes two stages: cluster formation and data collection. At the beginning of cluster formation stage, the information of 2*R* (*R* refers to the communication radius of a normal node and 2*R* is that of a cluster node) and $\bar{E}(r+1)$ is stored in each node's memory. During the cluster head formation stage, the weighted function of the CH selection probability is calculated firstly. Then, the management nodes make sure that the percentage of CHs is equal to the predefined priority percentage *p* in each round. At the same time, data collection also contains two substages: the stage of sending data and the stage of sending the information about the current status of energy consumption. EDFCM is a single-hop communication method to transmit the data to sink which means that the CHs communicate with sink directly.

### 3.2. MCR

In [22], Kumar et al. proposed an energy-efficient heterogeneous clustered (EEHC) scheme for WSNs. In this scheme, EEHC first calculates the optimal cluster numbers based on the side length of the sensing area and the total number of sensor nodes; then, according to the concept of SEP, the clustering algorithm contains two phases: the setup phase and the stable phase. In the setup phase, three different kinds of weighted probability formulas are defined for three kinds of the sensor nodes to elect their own CHs. After the CHs election, the other nodes choose a cluster and join in it. One CH takes the responsibility to transmit the data packets with a single-hop to sink node. The performance of the proposed EEHC system is better than LEACH and SEP in terms of reliability and lifetime. Based on their previous researches, in 2011, Kumar et al. proposed an energy-efficient multihop communication routing (MCR) protocol. MCR provides load balancing, lifetime enhancement, stability, and energy efficiency for the given HWSNs. MCR first calculates the optimal number of the CHs *k*_opt_ in the network based on the side length of the sensing area, node numbers, and the transmitter amplifier's multiple.

#### 3.2.1. The CH Election Weighted Probabilities

Protocol MCR uses both single-hop transmission and multihop transmission in the network. CHs are picked based on the same weighted probability formulas which are used in EEHC. Cluster member nodes communicate with the CH by using single-hop communication and CH communicates with the sink through multihop communication by choosing the proper CH nearest to the sink as the next hop. In MCR, normal nodes, advanced nodes, and super nodes are deployed randomly together in the sensing area to create the HWSN. The advanced nodes have more initial energy than the normal nodes, and the super nodes have more initial energy than the advanced nodes. The authors consider that *m*_0_ percentage of *m* nodes are super nodes which initially have *β* times more initial energy than the normal nodes and the *n*∗*m*∗(1 − *m*) fraction of total nodes are advanced nodes which initially have *α* times more initial energy than the normal nodes, and the remaining (1 − *m*) percentage of total nodes is normal nodes. *n* is the number of total sensor nodes. *E*_0_ is defined as the initial energy of the normal node; then, initial energy of each super node and each advanced node should be *E*_0_(1 + *β*) and *E*_0_(1 + *α*), respectively.

As described above, the total energy of the whole HWSN setting can be *E*_total_ = *nE*_0_(1 + *m*(*α* − *m*_0_(*α* − *β*))). As we can see from *E*_total_, the total initial energy is increased 1 + *m*(*α* − *m*_0_(*α* − *β*)) times compared with the homogeneous network. To make sure that the election of CHs of the network is stable, which means making these three kinds of nodes elect CHs separately, the new optimal epoch is defined as

(5) $$\begin{array}{c} \frac{1}{p_{\text{opt}}}\times \left(1+m\left(\alpha -m_{0}\left(\alpha -\beta \right)\right)\right). \end{array}$$

Then, the weighted probabilities of three kinds of nodes to become CHs are as follows:

(6) $$\begin{array}{c} p_{\text{normal}}=\frac{p_{\text{opt}}}{1+m\left(\alpha -m_{0}\left(\alpha -\beta \right)\right)},\\ p_{\text{advanced}}=\frac{p_{\text{opt}}}{1+m\left(\alpha -m_{0}\left(\alpha -\beta \right)\right)}\times \left(1+\alpha \right),\\ p_{\text{super}}=\frac{p_{\text{opt}}}{1+m\left(\alpha -m_{0}\left(\alpha -\beta \right)\right)}\times \left(1+\beta \right). \end{array}$$

By the above formulas, the authors can get threshold to elect the CHs for normal nodes, advanced nodes, and super nodes, respectively.

#### 3.2.2. Cluster Formation, Route Selection, and Data Transmission Phases

In cluster formation phase, non-CH nodes join the nearest CH simply by detecting the RSSI that depends on the received signal from the CHs. After the nodes have completely joined the clusters, a TDMA slot is needed for every cluster, and every CH node sends the TDMA slot to its member nodes to tell them when they can transmit the data. In route selection phase, a CH node aggregates the data from the member nodes and then transmits the data to the sink over a multihop path. Because the shortest path will have the lowest energy cost, a CH node chooses another CH as the next hop whose distance to sink is the shortest. In the data transmission phase, a CH node collects and aggregates the data from its member nodes in the fixed TDMA slot. After this, the CH transmits the data to the sink over the previously built multihop path in the route selection phase.

### 3.3. EEPCA

It is vital to reduce energy consumption and prolong network lifetime in designing an energy-efficient WSN. In [17], Peng et al. put forward a research on the existing cluster-based schemes for HWSNs and then proposed an energy-predicting clustering algorithm named energy-efficient prediction clustering algorithm (EEPCA). A CH in EEPCA is elected from the sensor nodes by using this algorithm mainly depending on energy consumption and communication cost; thus, the nodes with higher residual energy and lower communication cost are more likely to become a CH than the other nodes. Then, the energy of the network should be consumed uniformly. A prediction model for energy dissipation is also built for this algorithm to be more energy efficient.

#### 3.3.1. Calculation of the Distance between Nodes

The energy consumed by node *i* transmitting a message to node *j* is defined as *E*_*i*_^tran⁡^; at the same time, node *j* detects the received data strength with energy *E*_*j*,*i*_^rec^. If the distance between node *i* and node *j* is *d*_*i*,*j*_, then the relationship between *E*_*i*_^tran⁡^ and *E*_*j*,*i*_^rec^ is shown as follows:

(7) $$\begin{array}{c} E_{j,i}^{\text{rec}}=\frac{K}{d_{i,j}^{\theta }}\times E_{i}^{\mathrm{tran}}, \end{array}$$

where *K* is a constant and *θ* is the distance-energy gradient that changes from 1 to 6 depending on the application environment.

#### 3.3.2. Cluster Head Selection

Due to the burden of communications and processing various data, CH consumes a great deal of energy compared with the cluster member nodes. Thus, the nodes with more residual energy should have higher probability to become a CH. And it is the same for the other nodes to become a CH in the next round.

The probability *p*_*i*_ of becoming a CH of every node is changing in every round according to its current residual energy [14]. The authors first calculate the optimal number of cluster heads *K*_opt_, and then the proportion is

(8) $$\begin{array}{c} p_{\text{opt}}=\frac{K_{\text{opt}}}{N}, \end{array}$$

where *N* is the total number of nodes.

The average energy of the nodes within node *i*'s communication range is

(9) $$\begin{array}{c} w{\left(E\right)}_{i}=\frac{E_{i}}{\sum_{j-1}^{n}\left(E_{j}/n\right)}, \end{array}$$

where *n* is the number of nodes within node *i*'s communication range.

To predict the energy dissipation more precisely, the author divides the communication range of a node into two sublevels. Level one contains those nodes whose distance to the center node is smaller than *d*_0_, while level two contains those nodes whose distance to the center node is larger than *d*_0_, and *d*_0_ is a predefined constant.

If the number of nodes in level one is *m*_1_ and the number of nodes in level two is *m*_2_, then the average energy consumption of every round within every node's communication range is ${\bar{E}}_{i-\text{round}}$ and the predicted energy consumption of every node in every round is ${\bar{E}}_{\text{consume}}$, respectively.

Then, the communication cost factor is as follows:

(10) $$\begin{array}{c} w{\left(C\right)}_{i}=\frac{{\bar{E}}_{\text{consume}}}{{\bar{E}}_{i-\text{round}}}. \end{array}$$

After integrating *w*(*E*)_*i*_ and *w*(*C*)_*i*_, the probability of node *i* to be elected as a cluster head is

(11) $$\begin{array}{c} p_{i}=p_{\text{opt}}\left(aw{\left(E\right)}_{i}+bw{\left(C\right)}_{i}\right), \end{array}$$

where *a* + *b* = 1. Here, *a* and *b* will be set to be 0.5 while changing the other parameters in our later simulations to see the performance of EEPCA.

At last, a new threshold formula *T*_*i*_ for node *i* is similar to LEACH protocol, as shown in the following:

(12) $$\begin{array}{c} T\left(i\right)=\{\begin{array}{cc} \frac{p_{i}}{1-p_{i}\left(r\mathrm{mod}\left(1/p_{i}\right)\right)} & \\ \times \left[\begin{array}{c} \left(aw{\left(E\right)}_{i}+bw{\left(C\right)}_{i}\right)\\ +\;r_{s}\mathrm{div}\left(\frac{1}{p_{i}}\right)\\ \times \left(1-aw{\left(E\right)}_{i}+bw{\left(C\right)}_{i}\right) \end{array}\right], & \text{if}\;i\in G\\ 0, & \text{otherwise}, \end{array} \end{array}$$

where *r*_*s*_ is the number of rounds that a node fails to be selected as the cluster head.

## 4. Network Model

### 4.1. Node Deployment

**Figure 2:**
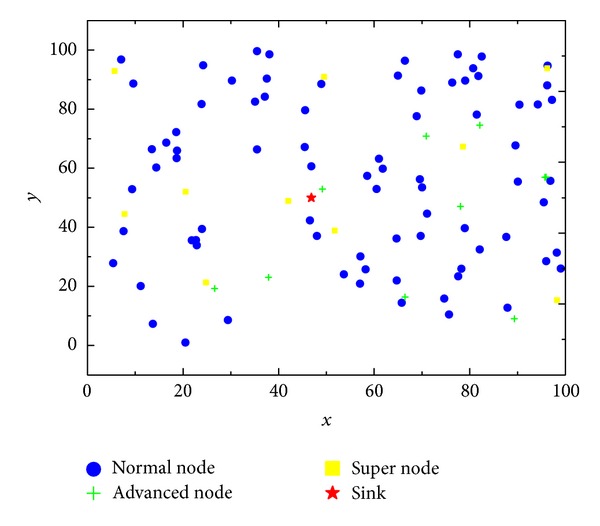
The network model with heterogeneity in three energy levels.

In this paper, different kinds of nodes with different energy but the same sensing radius and communication radius are deployed in the heterogeneous network. The basic model of the network is shown in Figure 2.

As we can observe from Figure 2, there are three types of nodes deployed in the network: normal nodes, advanced nodes, and super nodes, and they are shown in different colors and shapes. The difference between these three types of nodes is their initial energy. Sink is located at the center of the network, and the other sensor nodes are deployed randomly in the network area.

### 4.2. Energy Dissipation

**Figure 3:**
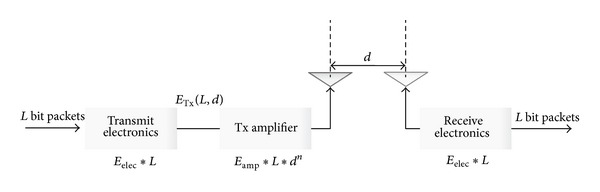
Radio energy dissipation model.

In our study, we use the similar energy dissipation model which is proposed in [2]. The radio energy dissipation model is illustrated in Figure 3. When a node transmits *L* bit message over a distance *d* to another node, the energy consumed by the radio is defined as

(13) $$\begin{array}{c} E_{\text{Tx}}\left(L,d\right)=\{\begin{array}{cc} L\times E_{\text{elec}}+L\times E_{\text{fs}}\times d^{2}, & \text{if}\;d<d_{0}\\ L\times E_{\text{elec}}+L\times E_{\text{fs}}\times d^{4}, & \text{if}\;d\geq d_{0}, \end{array} \end{array}$$

where *E*_fs_ and *E*_mp_ depend on the transmitter amplifier model, *d*_0_ is equal to $\sqrt{E_{\text{fs}}/E_{\text{mp}}}$, and the energy dissipation is defined as

(14) $$\begin{array}{c} E_{\text{Rx}}\left(L\right)=E_{\text{Rx}-\text{elec}}\left(L\right)=L\times E_{\text{elec}}. \end{array}$$

### 4.3. Simulation Setup

As shown in Table 2, sensor nodes are distributed in an area of 100 m∗100 m, and sink is located at the center of sensing area, and the number of nodes *N* is 100. The advanced node has *α* times more energy than the normal node, and the super node has *β* times more energy than the normal node. Priority percentage *p* is calculated theoretically according to the previous work. The fraction *m* is the fraction of the number of heterogeneous nodes of all nodes, and *m*_0_ is the fraction of super nodes of all the heterogeneous nodes. *R* is the sensing radius of single node and *r*_max⁡  _ is the total round of network or the running time of network. The parameters are the basis. We can change some of them to create the different simulation environments in our later experiments. There are three kinds of nodes with three energy levels. In simulations, we consider advanced node and super node in EDFCM to be type 0 and the normal node to be type 1. We also consider advanced node and super node to the same type of node in SEP. The details will be given in the following part. To evaluate the performance of the algorithms we introduced in this paper, we conduct extensive simulation experiments on Matlab.

## 5. Simulation and Performance Analysis

Simulations are run to compare the performance of the protocols in five scenarios in terms of the round of the first node dies and packets that sink receives. The former one refers to the stable period of the network which is very important in some occasions and the latter one refers to the monitoring ability which is also a critical factor in some WSN applications. Furthermore, we put forward another two scenarios to compare the lifetime of network.

**Figure 4:**
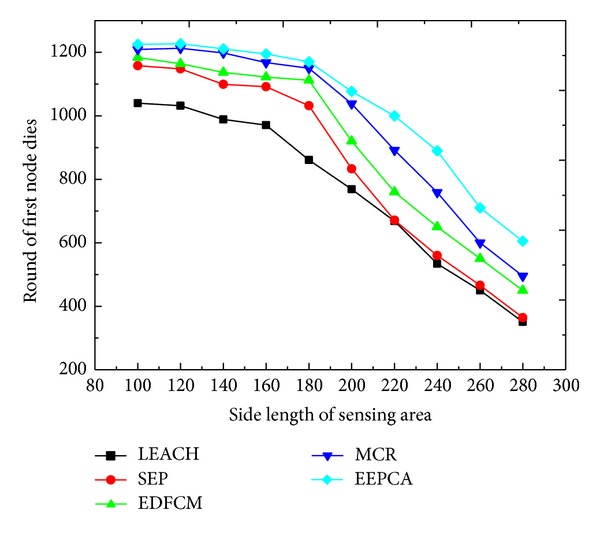
Round of first node dies with varying side length of sensing area.

**Figure 5:**
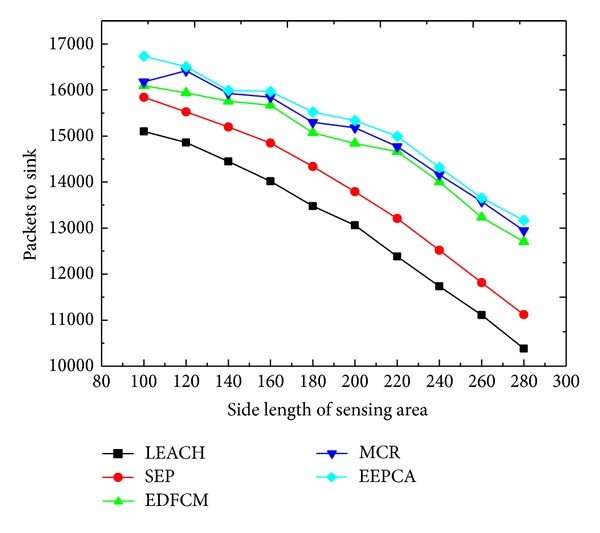
Packets to sink with varying side length of sensing area.

As shown in Figures 4 and 5, the packets that the sink receives and the round of the first node dies are decreasing with the increasing of the side length of the sensing area which is changing from 100 m to 280 m at the step of 20 m. Because of the increasing of side length, the density of nodes in the area is decreasing, which results in the distance between two nodes getting farther. One node has to consume more energy to transmit data to the neighbors. As a result, the energy dissipation of a single node and the network become higher. And the time when the first node dies becomes earlier correspondingly, which leads to the decrease of lifetime of the network as well as the number of packets received by the sink. We can also observe from the two figures that EEPCA, MCR, and EDFCM have better performance than the two former protocols, SEP and LEACH. EEPCA can make the energy of nodes uniformly consumed in the network, so it has higher energy efficiency than MCR and EDFCM. MCR utilizes a multihop way to transmit the data from CH to sink at the data transmission phase, and we know from other articles that most of the energy is used to transmit data from CH to sink. In MCR, three types of nodes have their own election probability to be stably selected as CHs, but MCR cannot uniformly consume the energy like EEPCA. EDFCM limits the number of CHs during the whole process; when the number of CHs is beyond the threshold, EDFCM randomly chooses some of CHs and turns them into a non-CH and when the number of CHs is below the threshold EDFCM also chooses some nodes with more energy to be CH. An energy prediction method is also introduced to predict every node's probability to decide which is most likely to be CH in the next round. However, EDFCM transmits the data from CH to sink by single-hop; thus, CH consumes more energy than MCR.

**Figure 6:**
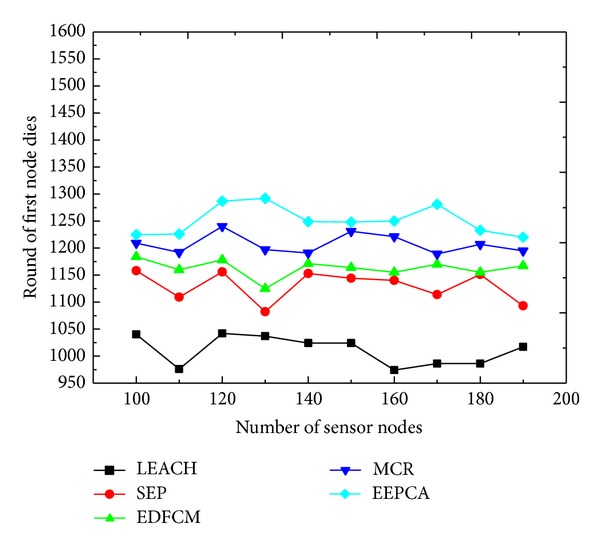
Round of first node dies with varying number of sensor nodes.

**Figure 7:**
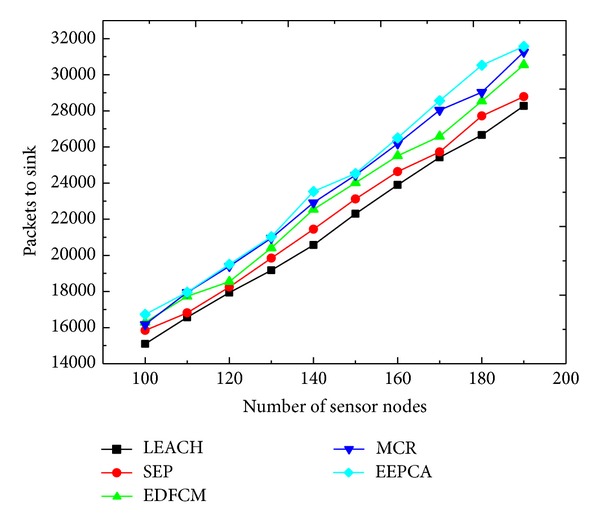
Packets to sink with varying number of sensor nodes.

In Figures 6 and 7, we change the number of nodes from 100 to 190 at the step of 10 to see how the five protocols work, and the other parameters remain the same as shown in Table 2. In Figure 6, we can observe that, with the increasing number of nodes, the time of the first node dies almost stays in the same levels among these five protocols. This is because even though the number of nodes increases, the average energy consumption of communication and data transmission in the cluster is almost the same and the average energy consumption in one node changes a little. We can also discover that EEPCA, MCR, and EDFCM have better performance in stable period than LEACH and SEP, because they can make the nodes dissipate their energy uniformly and balance the energy consumption to provide longer stable period.

In Figure 7, the reason why the packets sent to sink are increasing is that the increasing number of sensing nodes creates more sensing data, and, at the same time, they elect more CHs to transmit these data packets to sink. We can also observe from Figure 7 that EEPCA has better network monitoring quality than the other algorithms.

**Figure 8:**
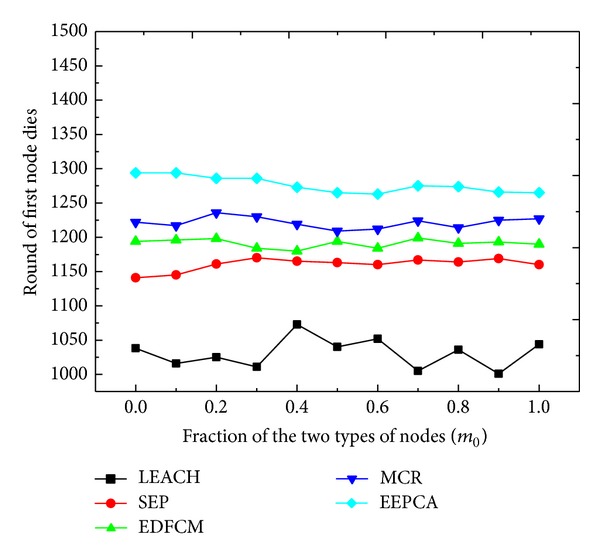
Round of first node dies with varying fraction of the two types of nodes.

**Figure 9:**
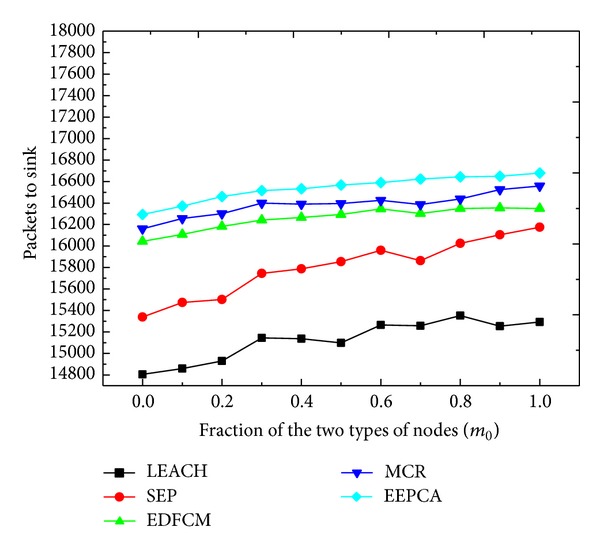
Packets to sink with varying fraction of the two types of nodes.

In Figures 8 and 9, we change the fraction *m*_0_ from 0 to 1 at the step of 0.1. *m*_0_ is the fraction of the super nodes in the total heterogeneous nodes, and the other parameters stay the same as shown in Table 2. We can observe from Figure 8 that the round of first node dies of these five protocols almost stays at the same level under different *m*_0_. This is because the first node dies usually occur to the normal node, and the fraction *m* does not change, which means that the number of normal nodes does not change. The energy consumed in communication and data transmission among the normal nodes remains almost the same, and the stable period almost stays at the same level. In Figure 9, with the increase of *m*_0_, the number of advanced nodes decreases, but the number of super nodes increases; at the same time, the total energy of heterogeneous nodes increases, which results in the increase of the energy of the entire network. With more energy, more nodes can survive for a longer time, which makes them transmit more packets to sink. That is why the number of packets sent to sink increases with the increase of *m*_0_. And, for the same reason we discussed above, EEPCA has the best network monitoring quality in these protocols, and the result shows that MCR is better than EDFCM.

**Figure 10:**
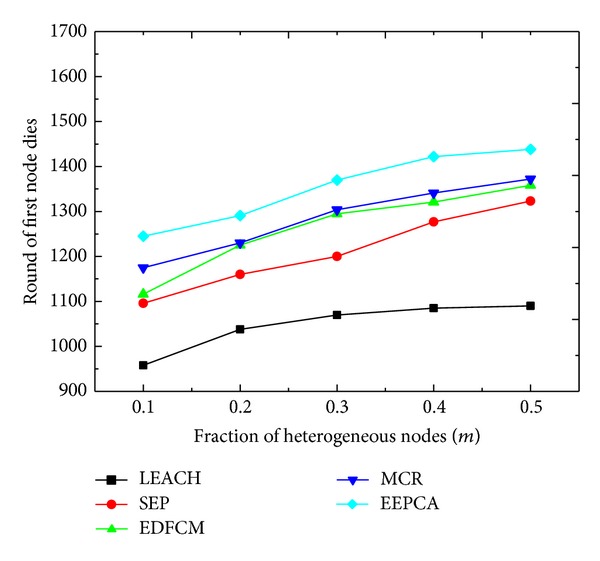
Round of first node dies with varying fraction of heterogeneous nodes.

**Figure 11:**
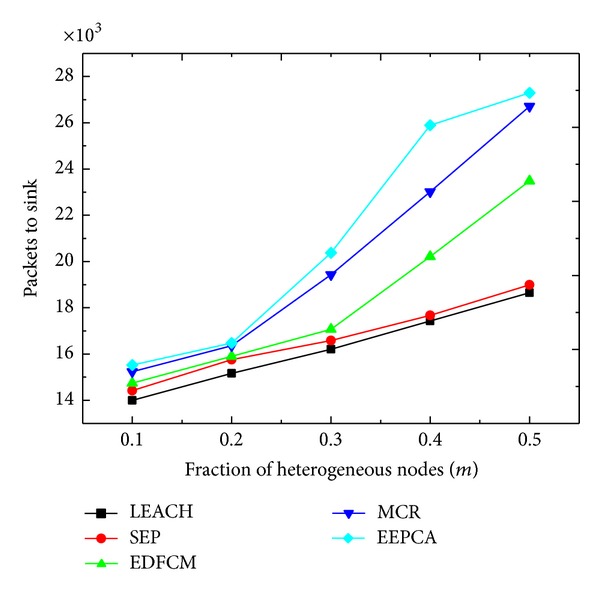
Packets to sink with varying fraction of heterogeneous nodes.

In Figures 10 and 11, we change the parameter *m* from 0.1 to 0.5 at the step of 0.1. *m* is the fraction of heterogeneous nodes and the other parameters stay the same as shown in Table 2. In Figure 10, we can observe that the round of first node dies increases slightly when *m* increases. This is because the increase of *m* means more heterogeneous and less normal nodes. These five protocols are all cluster-based, and the main idea of them is to elect the node which can best manage the cluster as a CH. So the node with more energy has the priority to be a cluster head. As we discussed above, the first node dies usually occur to the normal node, while the normal node mainly acts as the cluster member rather than a CH, which has a smaller energy consumption rate than the advanced node and super node. In all the five algorithms, the first node tends to die later with the increase of *m*. In Figure 11, it is apparent that the packets that the sink receives are increasing. Because the rising of *m* causes the total energy of network to rise, then the nodes have more time to collect and transmit data packets.

**Figure 12:**
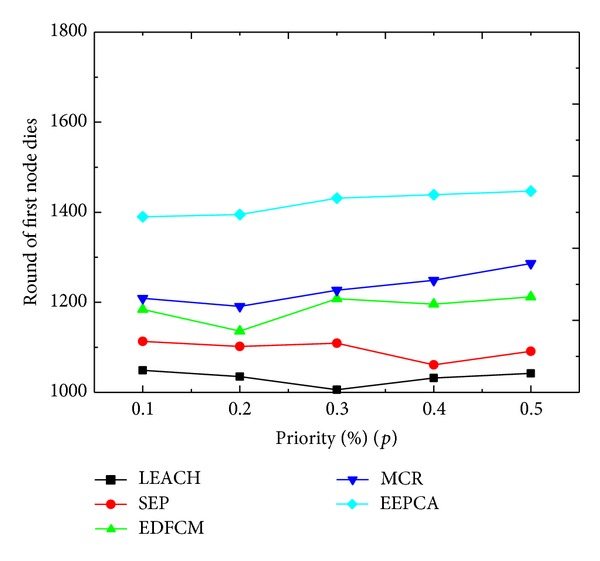
Round of first node dies with varying priority percentage.

**Figure 13:**
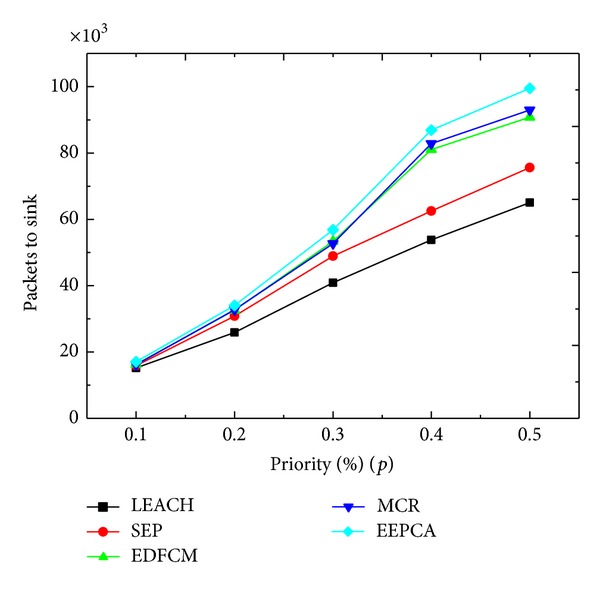
Packets to sink with varying priority percentage.

In Figures 12 and 13, we compare the performance under different values of *p* which is the priority percentage of CHs of all sensor nodes. As we can observe from the figure, the *p* changes from 0.1 to 0.5 at the step of 0.1. Round of first node dies of these five protocols does not change much under different values of *p*, while the packets that the sink receives increase a lot with the increasing of *p*. In Figure 12, the reason why the stable period of every protocol stays at almost the same level is that, at the beginning, cluster heads are mainly elected from the advanced nodes and super nodes because they have more energy to be capable of managing the clusters and they spend the most of the energy during the data gathering and transmitting phase. In contrast, most of the normal nodes have very little chance of being elected as a CH, and they need not consume that much energy correspondingly. Even though the increase of *p* leads to the increase of the probability of the normal nodes to be a cluster head, those heterogeneous nodes are still the main part of cluster heads. In Figure 13, we can observe that the amount of packets sent to sink is rising with the increase of priority percentage *p*, because the increase of *p* enables the nodes to elect more CHs to send the sensed data packets to sink.

**Figure 14:**
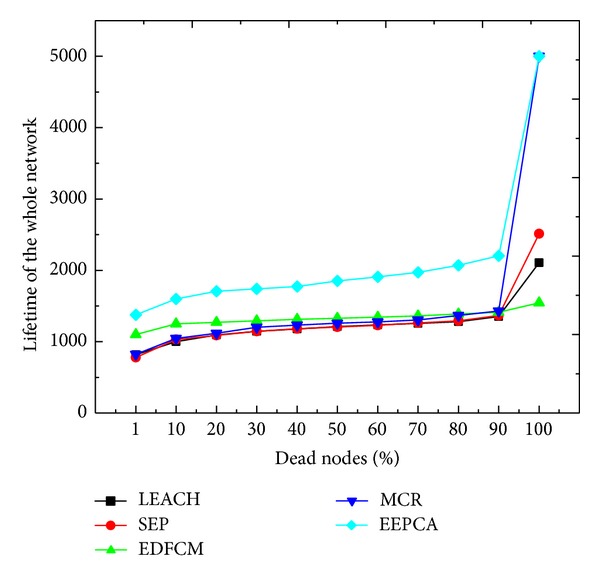
Lifetime of the whole network.

In Figure 14, we set *m* = 0, *m*_0_ = 0, *α* = 0, and *β* = 0; *m* is the fraction of total number of heterogeneous nodes of all nodes, *m*_0_ is the fraction of super nodes in the fraction *m*, *α* is the energy multiple which means that advanced node has *α* times more energy than the normal node, and *β* is the energy multiple which means that super node has *β* times more energy than the normal node. Figure 14 describes a homogeneous circumstance, and we can observe that heterogeneous cluster-based protocols have a longer lifetime than LEACH, and the former can also be applied in the homogeneous circumstance. Because EEPCA has a good ability of balancing energy consumption, it can achieve a longer stable period and lifetime no matter whether the network is homogeneous or heterogeneous. MCR uses both multihop and stable election to save energy. In fully distributed manner, EDFCM elects the CHs by using one-step energy consumption prediction, but a CH consumes much more energy when transmitting the packets to sink by single-hop, so its performance is not so much better than the former two protocols but much better than LECAH and SEP.

**Figure 15:**
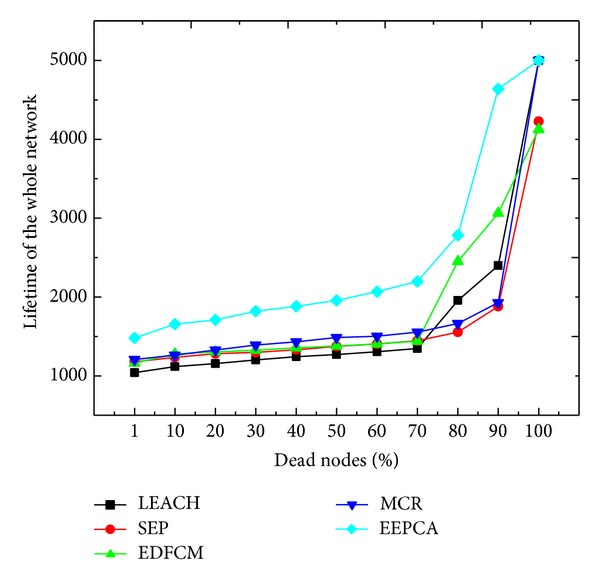
Lifetime of the whole network.

In Figure 15, *m* = 0.2, *m*_0_ = 0.5, *α* = 1, and *β* = 1.2, and there is no doubt that heterogeneous cluster-based protocols have the better performance, because these heterogeneous protocols have the ability to manage the clusters and their member nodes and can better balance the energy consumption of the nodes in the whole network.

## 6. Conclusions and Future Work

Simulation results show that the characteristics of HWSN algorithms are better than the homogeneous ones in terms of both the round of the first node dies and the number of packets sent to sink. As mentioned above, these heterogeneous cluster-based protocols have the ability to manage the clusters and their member nodes and can better balance the energy consumption of the nodes in the whole network. Moreover, the multihop path among CHs to sink is a very important concern to save energy during the data transmission. Our further work will mainly focus on how to further balance the energy consumption of every node by using the unequal clusters and on the moving heterogeneous sensor nodes. Furthermore, the energy whole problem is to be relieved in the network.

## Figures and Tables

**Table 1:** Recent proposed routing protocols.

Protocols	Prediction related	Data transmission	Evolutionary related	Energy efficiency
LEACH	No	Single-hop	No	Poor
SEP	No	Single-hop	No	Good
DEEC	Yes	Single-hop	No	Good
EDFCM	Yes	Single-hop	No	Good
REP	Yes	Single-hop	No	Good
EEPCA	Yes	Single-hop	No	Very good
HEED	No	Single-hop	No	Good
EHEED	No	Multihop	No	Good
EEHC	No	Single-hop	No	Very good
MCR	No	Multihop	No	Very good
EAERP	No	Single-hop	Yes	Good
ERP	No	Single-hop	Yes	Good
SAERP	No	Single-hop	Yes	Good

**Table 2:** Basic parameters used in simulations.

Parameter	Value
Sensing area	100 m ∗ 100 m
Sink location	(50 m, 50 m)
Number of nodes *N*	100
Priority percentage *p*	0.1
Initial energy of normal node	0.5 J
*α*	1.0
*β*	1.2
*m*	0.2
*m*_0_	0.5
*R*	25 m
Data packet size	4000 bits
*E*_elec_	50 nJ/bit
*E*_fs_	10 pJ/(bit∗m^2^)
*E*_mp_	0.0013 pJ/(bit∗m^4^)
*r*_max⁡_	5000
